# Loss of TIMM8A Inhibits Breast Cancer Progression Through Promoting HSPA9 Ubiquitination and Degradation via Competitive Interaction with RNF4

**DOI:** 10.3390/ijms27167382

**Published:** 2026-08-18

**Authors:** Yi Wu, Rujiao Liu, Jian Zhang, Shuiping Gao

**Affiliations:** 1 Key Laboratory of Breast Cancer in Shanghai, Fudan University Shanghai Cancer Center, Shanghai 200032, China; wuyi_smile@163.com; 2 Department of Oncology, Shanghai Medical College, Fudan University, Shanghai 200032, China; 06301010252@fudan.edu.cn; 3 Phase I Unit, Fudan University Shanghai Cancer Center, Shanghai 200032, China

**Keywords:** TIMM8A, HSPA9, RNF4, ubiquitination, breast cancer

## Abstract

To investigate the role and underlying mechanisms of TIMM8A in breast cancer progression. The expression of TIMM8A in breast cancer tissues and cell lines was assessed. Functional assays were performed to evaluate the effects of TIMM8A knockdown on proliferation, metastasis, and apoptosis in BT549 and MCF-7 cells. Co-immunoprecipitation (Co-IP) and ubiquitination assays were used to examine the interaction between TIMM8A and HSPA9, as well as the involvement of the E3 ubiquitin ligase RNF4. PI3K/AKT pathway activity was assessed by Western blotting. In vivo tumor growth was evaluated using a xenograft mouse model. TIMM8A was significantly upregulated in breast cancer tissues and cell lines, and high TIMM8A expression correlated with poor prognosis. TIMM8A knockdown suppressed proliferation and metastasis while inducing apoptosis in vitro. Mechanistically, TIMM8A interacted with HSPA9 and maintained its protein stability by inhibiting RNF4-mediated ubiquitination and degradation, thereby sustaining PI3K/AKT signaling activation. Conversely, TIMM8A depletion reduced HSPA9 stability and attenuated the pathway. Rescue experiments confirmed that HSPA9 overexpression restored PI3K/AKT activation and reversed TIMM8A-knockdown-induced malignant suppression. In vivo, TIMM8A silencing efficiently inhibited tumor growth, accompanied by reduced HSPA9 and Ki-67 expression and decreased PI3K/AKT phosphorylation. TIMM8A promotes breast cancer progression through the TIMM8A/HSPA9/PI3K/AKT regulatory axis, highlighting TIMM8A as a promising prognostic biomarker and potential therapeutic target for breast cancer.

## 1. Introduction

Breast cancer ranks first in both incidence and mortality among women worldwide. According to 2022 global statistics, breast cancer accounts for 23.8% of all female cancers and 15.4% of cancer-related deaths, posing a serious threat to women’s health worldwide [1]. Compared with other tumor types, the diagnosis and treatment of breast cancer have always been at the forefront of precision medicine. Clinically, based on the expression levels of estrogen receptor (ER), progesterone receptor (PR), human epidermal growth factor receptor 2 (HER2), and the proliferation marker Ki-67, breast cancer is classified into four major subtypes: Triple-negative breast cancer (TNBC), HER2-positive, Luminal A and Luminal B [2]. Although a great achievement has been made in breast cancer therapy, such as DS-8201 [3,4,5,6] and Dato-ADC [7], which have significantly improved the survival of patients with HER2-positive or TNBC, patients with advanced or distant metastatic disease still face formidable challenges [8]. Therefore, continuous exploration of novel therapeutic targets remains an indispensable part of breast cancer management. Given the high heterogeneity and adaptability of advanced breast cancer, there is an urgent need to discover new targets and build a more powerful “arsenal” to bring longer-lasting benefits to patients.

Mitochondria serve as crucial energy-metabolizing organelles and participate in various tumor biological processes through multiple pathways [9]. Previous studies have shown that mitochondrial DNA copy number (mtDNAcn) is closely associated with breast cancer and is considered a potential biomarker for cancer development [10,11,12]. The accumulation of mitochondrial DNA mutations can lead to oxidative phosphorylation dysfunction and increase the production of reactive oxygen species (ROS), thereby promoting tumor initiation and progression [13]. Moreover, mitochondria-related proteins also play an important role in the invasion and metastasis of breast cancer, such as the elevated expression of the pro-fission protein DRP1, while the expression of the pro-fusion proteins MFN1, MFN2, and OPA1 is reduced. This expression imbalance results in mitochondrial fragmentation, which in turn facilitates tumor invasion and metastasis [14,15].

TIMM8A (translocate of inner mitochondrial membrane 8a) is a protein-coding gene located on the X chromosome and was first reported in 2003 [16]. The TIMM8A protein exists in the mitochondrial intermembrane space, where it can form a complex with Tim13 to facilitate the translocation of nuclear-encoded precursor proteins into the mitochondrial inner membrane [17]. Initial studies revealed that mutations in the TIMM8A gene can lead to a rare X-linked recessive neurodegenerative disorder known as Mohr–Tranebjærg syndrome (MTS). Nowadays, elevated expression of TIMM8A is recognized to be closely correlated with poor prognosis in patients with breast and endometrial cancers [18]. Mechanistic studies have shown that TIMM8A is negatively regulated by the tumor-suppressive miR-10b-5p and may promote breast cancer cell proliferation, migration, and invasion, potentially through the activation of signaling pathways such as NF-κB [19]. In summary, TIMM8A plays an important role in breast cancer, though its precise molecular mechanism remains unclear. Therefore, this study aims to further validate the function of TIMM8A and focus on elucidating its underlying molecular mechanism.

## 2. Results

### 2.1. *TIMM8A* Is Highly Expressed in Breast Cancer and Associated with Poor Prognosis

**Figure 1:**
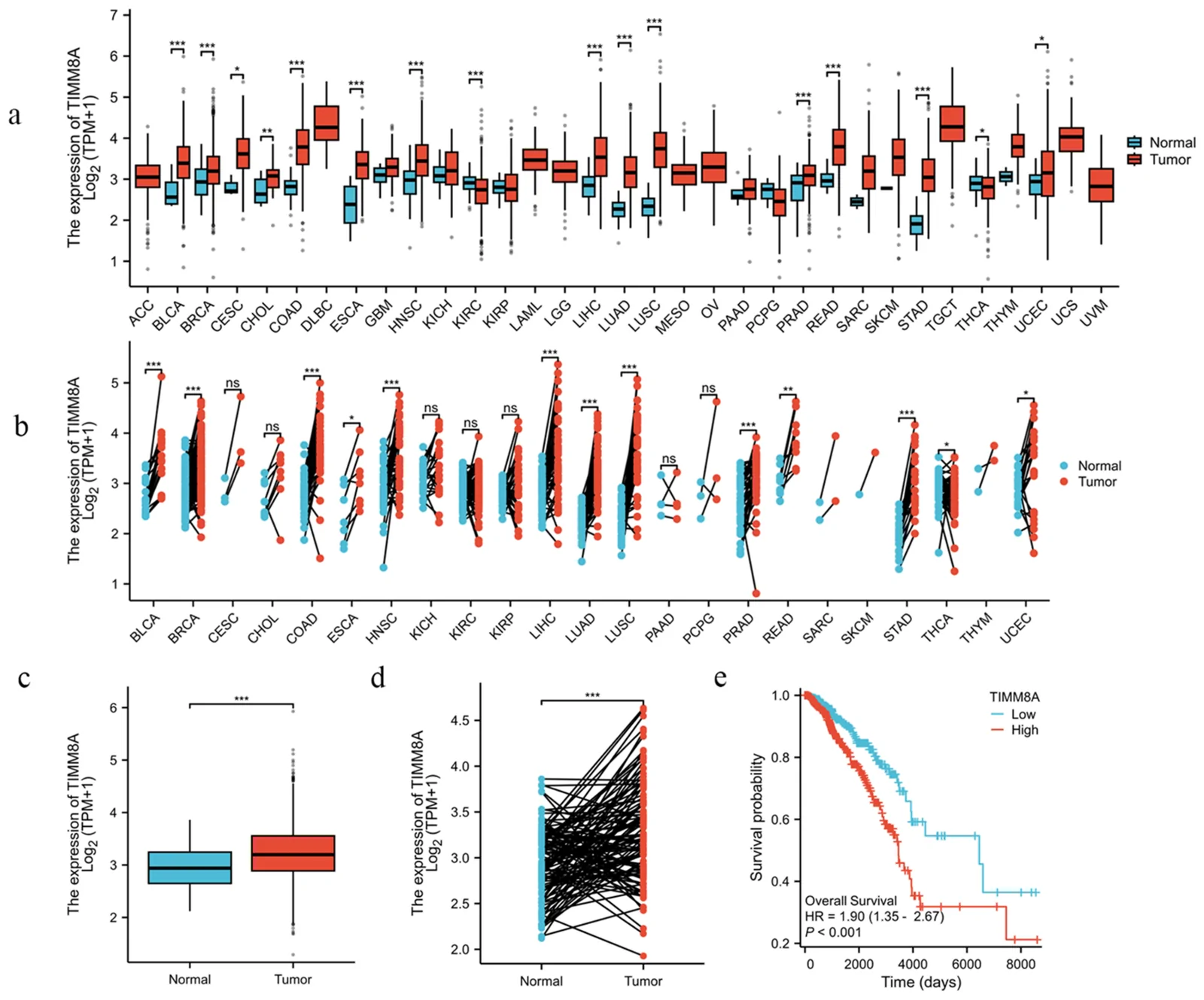
Expression of TIMM8A in different types of tumors and poor OS survival in BRCA patients according to the TCGA database. Expression of TIMM8A in (**a**) unpaired and (**b**) paired samples from the TCGA database. Expression of TIMM8A in (**c**) unpaired and (**d**) paired samples from the TCGA-BRCA database. (**e**) The correlation between TIMM8A expression and survival prognosis of breast cancer in TCGA. ns, *p* ≥ 0.05; * *p* < 0.05; ** *p* < 0.01; *** *p* < 0.001.

First, we performed differential expression analysis of TIMM8A across pan-cancer and found that TIMM8A is highly expressed in 20 malignant tumor types, including bladder urothelial carcinoma (BLCA), breast cancer (BRCA), esophageal carcinoma (ESCA) and colon adenocarcinoma (COAD) (*p* < 0.05, Figure 1a). Furthermore, in paired pan-cancer samples, TIMM8A expression was also significantly elevated in multiple tumor types (Figure 1b). In both paired and unpaired samples from the TCGA-BRCA database (Figure 1c,d), TIMM8A expression levels were significantly higher in tumor tissues than in normal tissues (*p* < 0.05). Subsequent survival analysis showed that patients with high TIMM8A expression had a poorer prognosis than those with low TIMM8A expression (Figure 1e).

**Figure 2:**
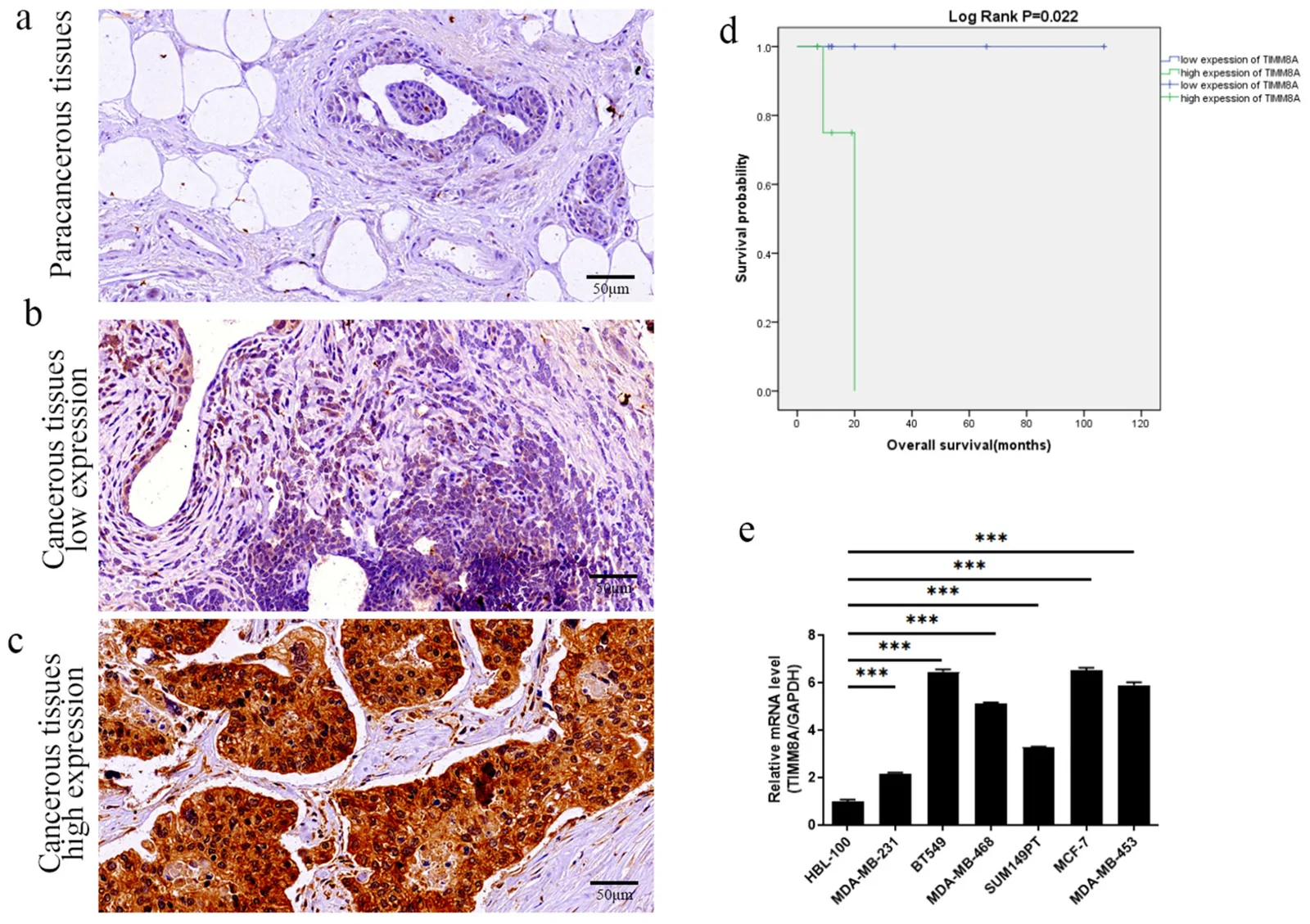
Expression of TIMM8A in our samples. (**a**–**c**) Representative TIMM8A IHC staining is presented in the large (400× magnification) images. Paracancerous tissues (**a**) and cancerous tissues (**b**) represent low TIMM8A expression, and (**c**) represents high TIMM8A expression. (**d**) Kaplan–Meier analysis of OS between low and high expression of TIMM8A in patients. (**e**) The mRNA level of TIMM8A in several breast cancer cell lines by qRT-PCR. *** *p* < 0.001.

Then, we collected pairs of adjacent normal and breast cancer tissues from 52 patients for immunohistochemistry. After excluding cases with tissue detachment or missing survival data, 31 cancer tissues and 51 adjacent tissues were finally included in the survival analysis. We found that TIMM8A expression was higher in cancer tissues than in adjacent normal tissues (Figure 2a–c), and paracancerous tissues exhibited extremely lower expression of TIMM8A, and the difference between cancer and adjacent normal tissues was statistically significant (*p* < 0.001). Based on the immunohistochemistry (IHC) scoring system, 51.6% of patients exhibited high expression and 48.4% exhibited low expression in cancer tissues (Table 1). Further Kaplan–Meier survival analysis based on survival data revealed that high expression of TIMM8A was associated with poor overall survival in breast cancer (Figure 2d). In addition, we examined TIMM8A expression in breast cancer cell lines and found that its expression was elevated in breast cancer cell lines compared with normal HBL-100 cells, with particularly high expression in BT-549 and MCF-7 cells (Figure 2e).

### 2.2. Loss of *TIMM8A* Inhibits the Proliferation, Migration, and Invasion of Breast Cancer Cells

**Figure 3:**
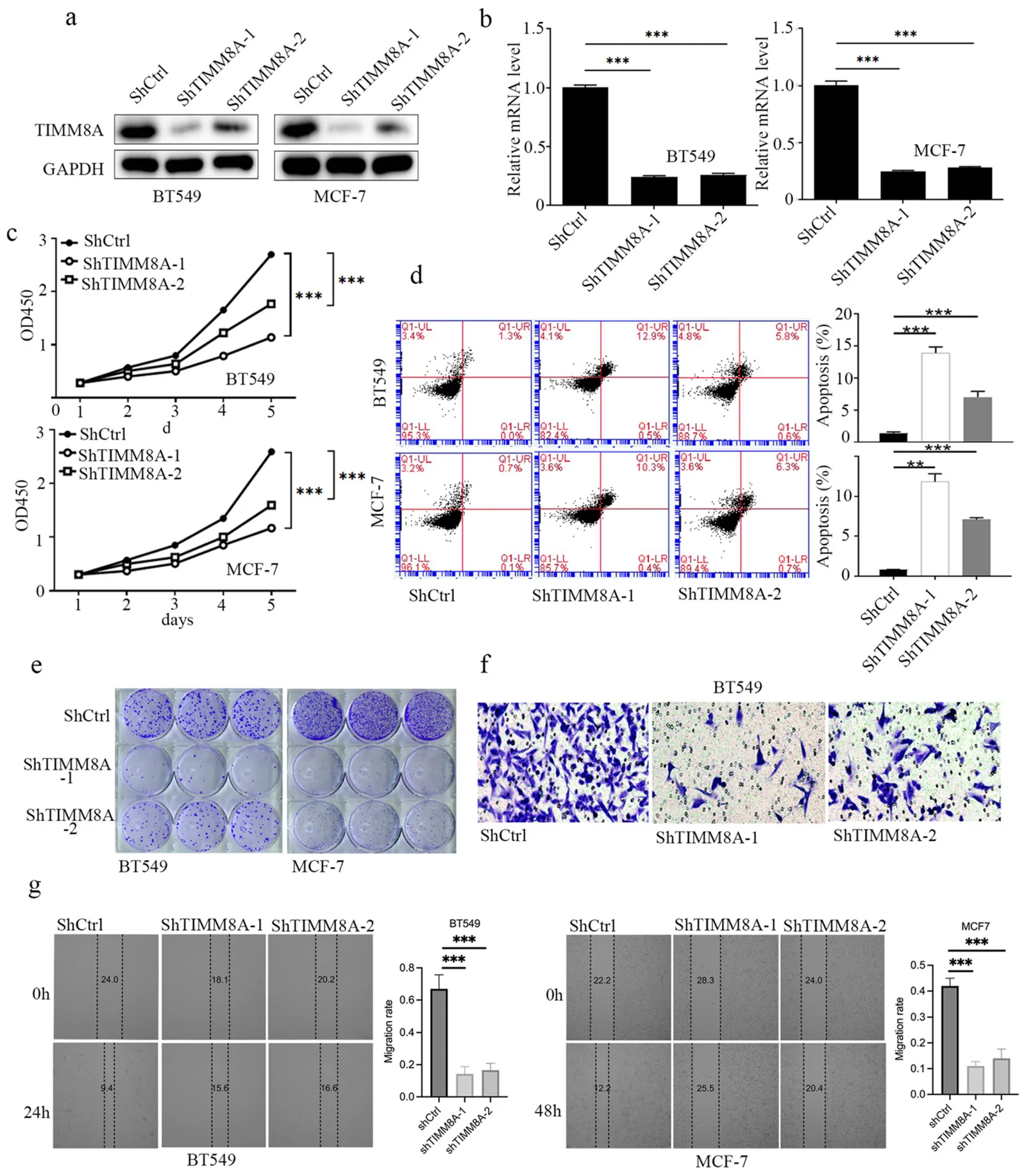
Downregulating TIMM8A suppresses the biological functions of tumor cells. (**a**) Western blot analysis of TIMM8A by shRNAs in BT549 and MCF-7 cells. (**b**) The mRNA level of TIMM8A in BT549 and MCF-7 cells by shRNA. (**c**) The proliferation of two stably knocked-down cell lines. (**d**) Apoptosis induced by loss of TIMM8A. (**e**) The photos of colony formation of two stable cell lines. (**f**) The migration of BT549 stable downregulation cells using the transwell assay. (**g**) Wound healing assay and statistical analysis of stably downregulated cell lines. Representative images are presented, ** *p* < 0.05; *** *p* < 0.001.

Based on the above results, we constructed stable knockdown cell lines in BT-549 and MCF-7 cells by sh-RNA (Figure 3a,b). In further functional assays, we found that when TIMM8A was knocked down, cell proliferation ability was significantly reduced, as measured by the CCK-8 assay (Figure 3c), while apoptosis was increased compared with the control (Figure 3d). In addition, colony formation assays showed that colony-forming ability decreased after TIMM8A knockdown (Figure 3e). Furthermore, transwell migration assays and wound healing assays were performed, and we found that loss of TIMM8A significantly inhibited the migratory capacity of breast cancer cells compared with the control group (Figure 3f,g).

### 2.3. Loss of *TIMM8A* Promotes *RNF4*-Mediated Ubiquitination and Degradation of *HSPA9*

**Figure 4:**
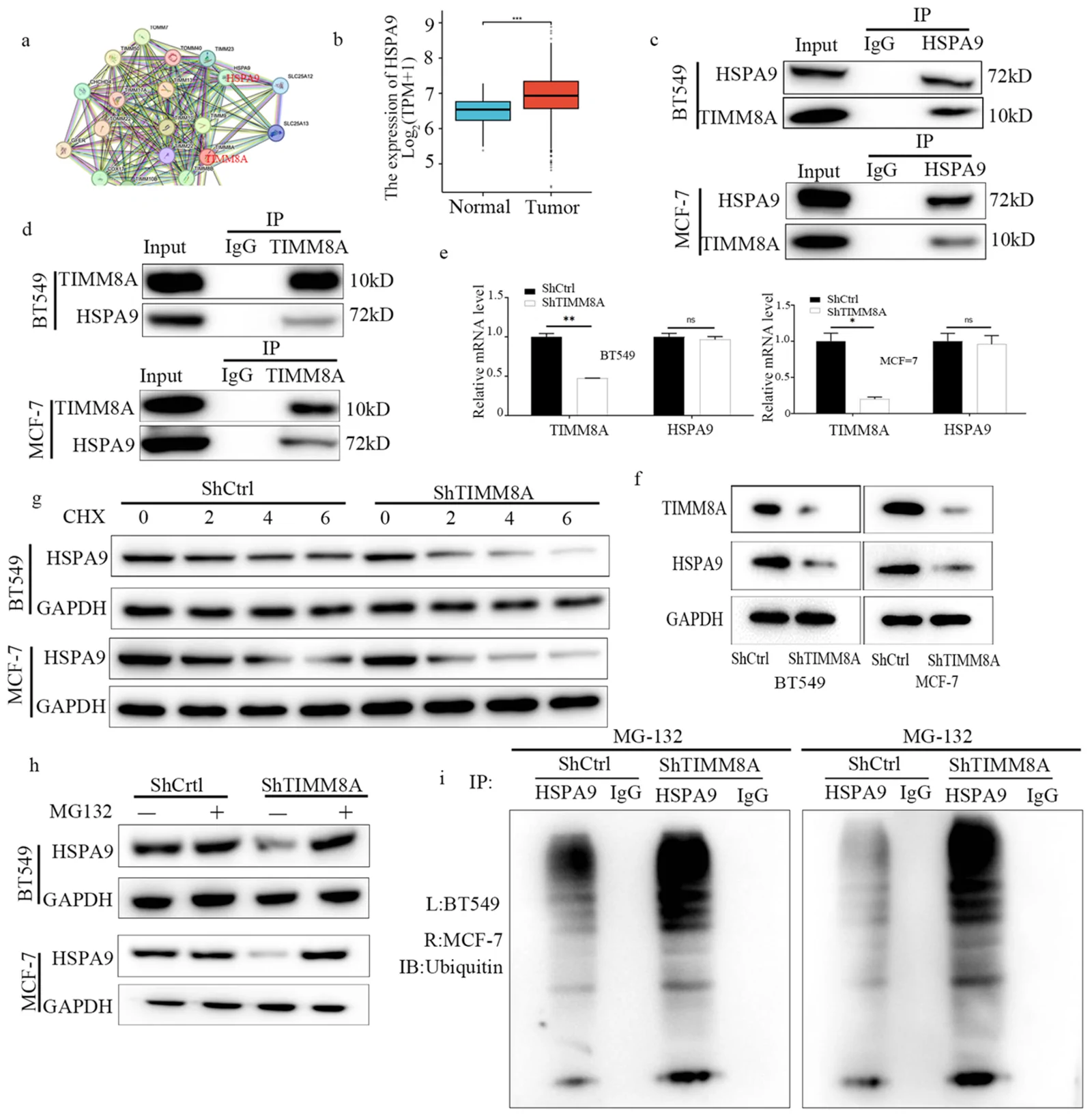
Co-immunoprecipitation (co-IP) assays to test the interaction between TIMM8A and HSPA9. (**a**) The protein–protein interaction network diagram showed the results of predicting proteins that might interact with TIMM8A using the STRING online tool. (**b**) The HSPA9 level between normal and breast cancer samples according to the TCGA database. (**c**,**d**) The interaction between TIMM8A and HSPA9 was detected by Co-IP. (**e**) The mRNA level of TIMM8A and HSPA9 in BT549 and MCF-7 stable sh-RNA cell lines. (**f**) The protein level of TIMM8A and HSPA9 in BT549 and MCF-7 stable sh-RNA cell lines. (**g**) BT549 and MCF-7 stable sh-RNA cell lines were treated with CHX, and the protein levels of HSPA9 were detected by Western blot at 0, 2, 4, and 6 h. (**h**) HSPA9 protein levels were detected by Western blot in two stable sh-RNA cell lines treated with or without MG-132. (**i**) Co-IP was performed to detect the ubiquitination level of the HSPA9 protein in two stable sh-RNA cell lines following MG-132 treatment. * *p* < 0.05; ** *p* < 0.01; *** *p* < 0.001; ns, *p* ≥ 0.05.

To further investigate the mechanism, we predicted TIMM8A-interacting proteins using the STRING website (https://cn.string-db.org, version: 12.0: since 26 July 2023), and found a high protein–protein interaction score between TIMM8A and HSPA9 (Figure 4a). Meanwhile, analysis of the TCGA database revealed that HSPA9 is significantly highly expressed in breast cancer tissues (Figure 4b). To verify the relationship between TIMM8A and HSPA9, we performed co-immunoprecipitation (Co-IP) assays in BT549 and MCF-7 stable cell lines, and the results confirmed that TIMM8A and HSPA9 indeed interact with each other (Figure 4c,d). We also observed that HSPA9 mRNA levels did not change significantly after TIMM8A was knocked down, whereas HSPA9 protein levels were markedly decreased (Figure 4e,f). Given that TIMM8A only affects HSPA9 protein levels, we speculated that this regulation might be related to post-translational modifications.

It is well known that ubiquitination is an important post-translational modification (PTM) mediated by ubiquitin-activating enzymes (E1), ubiquitin-conjugating enzymes (E2), and ubiquitin ligases (E3) [20]. The ubiquitin–proteasome system (UPS) regulates various cellular processes by directing protein degradation and is involved in multiple pathophysiological mechanisms of breast cancer, closely associated with the expression or function of target proteins [21]. Treatment with the protein synthesis inhibitor CHX in BT549 and MCF-7 sh-TIMM8A stable cell lines showed that TIMM8A knockdown accelerated the degradation of the HSPA9 protein, indicating that TIMM8A knockdown reduces HSPA9 protein stability (Figure 4g). Furthermore, treating BT549 and MCF-7 stable knockdown cell lines with proteasome inhibitor MG-132 reversed the inhibitory effect of sh-TIMM8A on HSPA9 (Figure 4h), and the ubiquitination level of HSPA9 in sh-TIMM8A cells was significantly increased compared to the control group (Figure 4i). Together, these results indicate that the downregulation of HSPA9 by sh-TIMM8A is mediated through the ubiquitination pathway.

**Figure 5:**
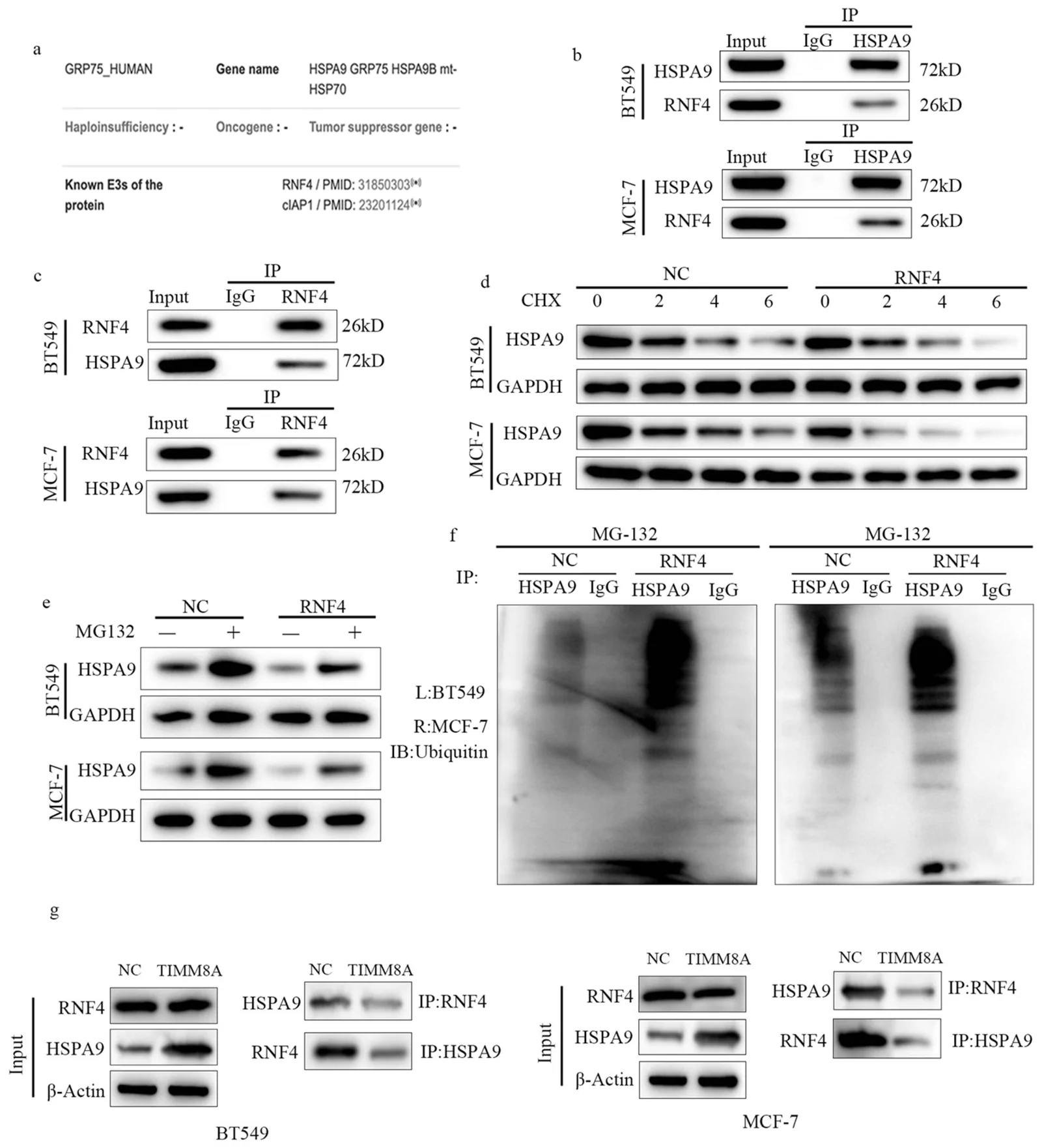
RNF4 is involved in the ubiquitination of HSPA9. (**a**) RNF4 is a known E3 ligase of HSPA9 by the Degpred online tool. (**b**,**c**) The interaction between HSPA9 and RNF4 was detected by Co-IP. (**d**) BT549 and MCF-7 RNF4-overexpressing cell lines were treated with CHX, and the protein levels of HSPA9 were detected by Western blot at 0, 2, 4, and 6 h. (**e**) HSPA9 protein levels were performed by Western blot in RNF4 overexpression cell lines treated with or without MG-132. (**f**) The ubiquitination level of the HSPA9 protein in two stable RNF4 overexpression cell lines following MG-132 treatment. (**g**) The interaction of RNF4 and HSPA9 weakens in TIMM8A overexpression stable cell lines.

To further identify the upstream E3 ligase regulating HSPA9 ubiquitination, we used the Degpred database (http://degron.phasep.pro/ (accessed on 1 July 2026), 2022-05-11version) for prediction and found that RNF4 might be the E3 ligase for HSPA9 ubiquitination (Figure 5a). Subsequently, we established RNF4-overexpressing stable cell lines in BT549 and MCF-7 cells and verified the interaction between HSPA9 and RNF4 by Co-IP assays (Figure 5b,c). Treatment with CHX in RNF4-overexpressing cells showed that increased RNF4 expression accelerated HSPA9 protein degradation (Figure 5d); conversely, treatment with MG-132 reversed the RNF4-mediated degradation of HSPA9, and RNF4 increased the ubiquitination level of HSPA9 (Figure 5e,f). Then, we established TIMM8A high-expression cell lines BT549 and MCF-7, and we found that TIMM8A overexpression enhances the stability of HSPA9, while the interaction between RNF4 and HSPA9 is reduced by conducting CO-IP experiments (Figure 5g). Based on these findings, we speculate that TIMM8A may competitively bind to HSPA9, thereby inhibiting RNF4-mediated ubiquitination and degradation of HSPA9, thus promoting the malignant progression of breast cancer.

### 2.4. *TIMM8A* Promoted Breast Cancer Progression by Upregulating *HSPA9*

**Figure 6:**
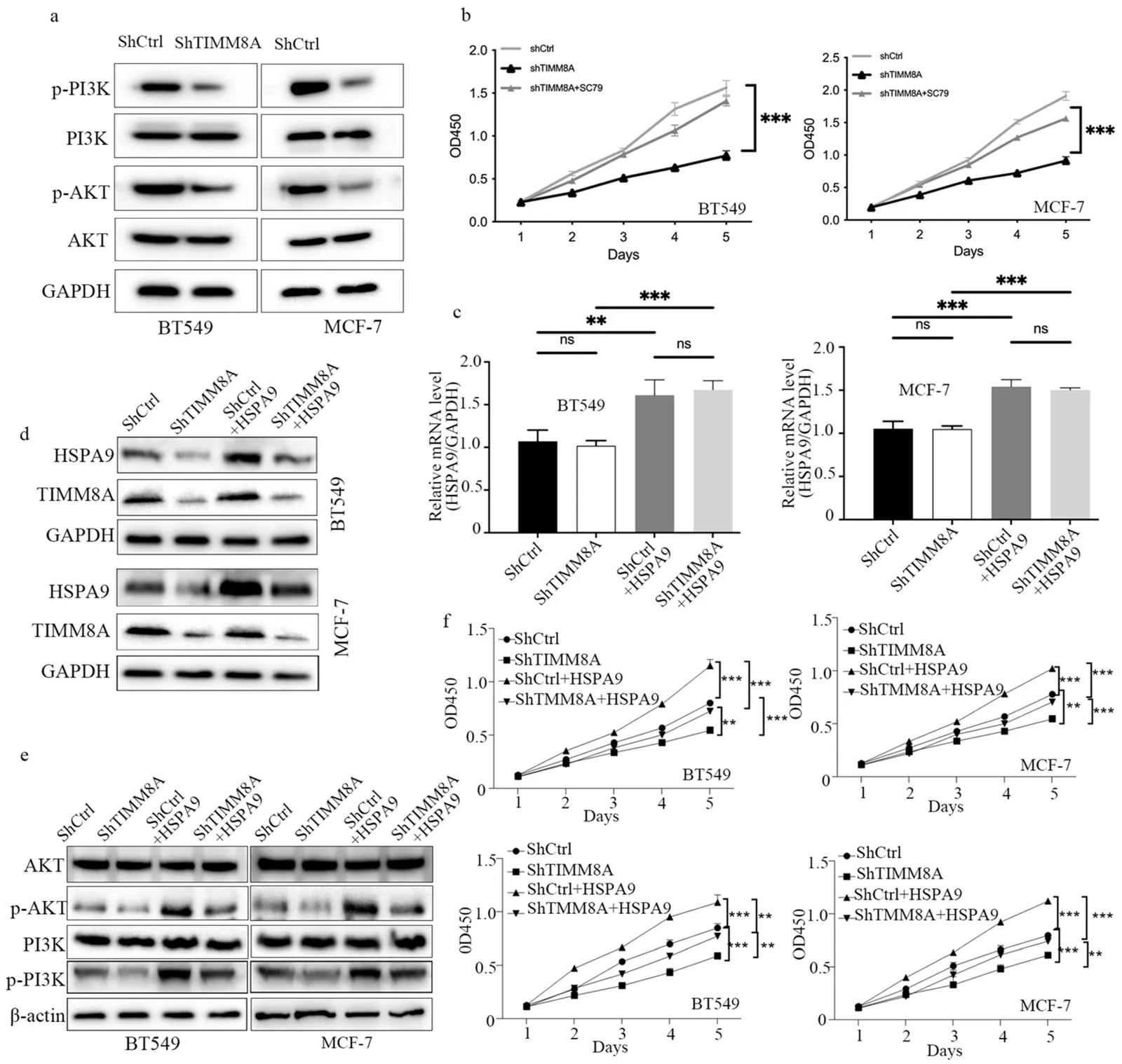
TIMM8A promoted breast cancer progression by upregulating HSPA9. (**a**) The PI3K/AKT signal pathway was inhibited in sh-TIMM8A breast cancer cells. (**b**) The proliferation of sh-TIMM8A cells was partly rescued by AKT agonist SC79. (**c**) The mRNA level of HSPA9 in each group with or without HSPA9 rescued. (**d**) The protein expression of HSPA9 in each group with or without HSPA9 rescued. (**e**) The PI3K/AKT signal pathway in each group with or without HSPA9 rescued. (**f**) The proliferation of two stably knocked down cell lines with or without HSPA9 rescued. ns, *p* ≥ 0.05; ** *p* < 0.01; *** *p* < 0.001.

Previous studies have reported that HSPA9 activates the PI3K/AKT signaling pathway and promotes malignant progression of breast cancer [22]. Based on the regulatory relationship of TIMM8A on HSPA9 expression, we hypothesized that TIMM8A may activate the AKT pathway through HSPA9-mediated signaling. Then, we found that silencing TIMM8A significantly inhibited the phosphorylation levels of PI3K and AKT by Western blot (Figure 6a). However, treatment of knockdown cells with the AKT activator SC79 markedly reversed the inhibitory effect of sh-TIMM8A cells as measured by CCK-8 proliferation assays (Figure 6b), suggesting that TIMM8A silencing blocks the activation of the PI3K/AKT pathway. To further clarify the regulatory role of HSPA9 in TIMM8A-mediated PI3K/AKT pathway activation and malignant phenotypes of breast cancer cells, HSPA9 was overexpressed in TIMM8A-silenced cells (Figure 6c,d). The results revealed that overexpression of HSPA9 effectively restored the PI3K/AKT phosphorylation suppressed by TIMM8A knockdown (Figure 6e). Subsequent CCK-8 assays further confirmed that overexpression of HSPA9 significantly reversed the inhibitory effects of sh-TIMM8A cells on the proliferation (Figure 6f). These results indicate that TIMM8A activates the PI3K/AKT signaling axis by upregulating HSPA9, thereby promoting the malignant progression of breast cancer.

### 2.5. *TIMM8A/HSPA9/PI3K-AKT* Axis Promoted Breast Cancer Progression In Vivo

**Figure 7:**
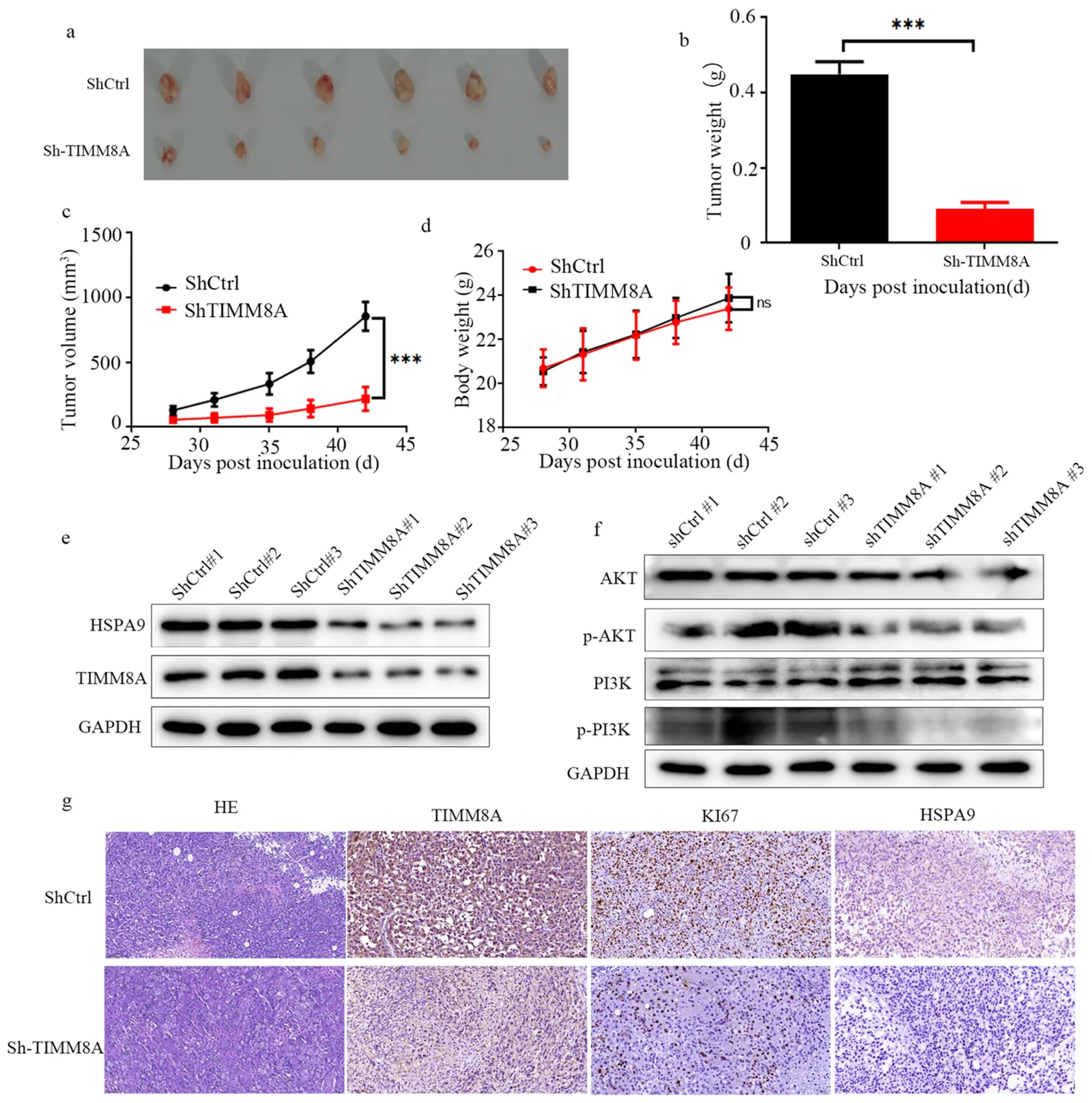
TIMM8A promoted tumor growth in vivo by upregulating HSPA9. (**a**) Nude mice were orthotopically injected with BT549 stable TIMM8A knockdown cells, and solid tumors were removed from nude mice after nude mice were sacrificed. (**b**) The weight of tumors. (**c**) The tumor growth was detected at different time points. (**d**) The body weight of mice in two knockdown groups. (**e**) The TIMM8A and HSPA9 expression in three representative tumors from each group. (**f**) The PI3K/AKT signal pathway in two knockdown groups. (**g**) The levels of Ki-67, TIMM8A and HSPA9 proteins in tumor tissues were assessed by immunohistochemical staining. *** *p* < 0.001.

To further investigate the role of TIMM8A in vivo, we orthotopically implanted BT549-modified cells (sh-control or sh-TIMM8A) into mouse mammary glands. The results showed that the tumor growth rate was significantly slower (Figure 7a,b) and the final tumor volume was markedly decreased (Figure 7c) in the sh-TIMM8A group compared to the control group, while there was no significant difference in body weight between the two groups (Figure 7d), suggesting that TIMM8A knockdown suppresses tumor growth in vivo without affecting the weight of mice. And then total tumor protein was extracted from three mice in each group for Western blot analysis. The results demonstrated that TIMM8A and HSPA9 expression was downregulated, and the phosphorylation levels of PI3K and AKT were also inhibited in the TIMM8A knockdown group (Figure 7e,f). Immunohistochemical staining further revealed that the expression levels of both HSPA9 and the proliferation marker Ki-67 were reduced in the TIMM8A knockdown group compared to the corresponding group. Collectively, these findings confirm that the TIMM8A/HSPA9/PI3K-AKT signaling axis regulates tumor growth in vivo, which is highly consistent with the results obtained in vitro.

## 3. Discussion

Breast cancer ranks first in both incidence and mortality among female malignant tumors and has become a leading cause seriously threatening women’s health [23]. Although remarkable achievements have been made in the clinical practice of breast cancer in recent years, especially the advent of antibody–drug conjugates (ADCs) that has brought new breakthroughs in clinical treatment [24], the overall survival of patients with advanced breast cancer remains unsatisfactory. Identifying and exploring novel therapeutic targets is still the core requirement in current breast cancer research. Therefore, in-depth exploration of potential key targets in breast cancer and clarification of their mechanisms possess important theoretical value and clinical significance.

TIMM8A was originally identified to be associated with Mohr–Tranebjaerg syndrome (MTS), a rare neurodegenerative disorder [16]. During programmed cell death, DDP/TIMM8A released in a Bax/Bak-dependent manner promotes Drp1-mediated mitochondrial fission and mitophagy [25]. Multiple studies have confirmed that TIMM8A is significantly upregulated in breast cancer tissues and is closely associated with poor patient prognosis [26,27]. Mechanistic studies have demonstrated that the TIMM8A-TIMM13 complex plays a critical role in the proliferation and growth of lung cancer cells, suggesting that TIMM8A may serve as a potential therapeutic target for lung cancer [28]. In addition, TIMM8A expression is correlated with tumor immune cell infiltration [29]; TIMM8A downregulation reduces NF-κB p65 expression, upregulates pro-apoptotic proteins, and modulates the expression of EMT-related proteins [19]. Meanwhile, the methylation level of the seventh CpG site in the TIMM8A gene may be related to patient prognosis, and CTD database analysis indicates that drugs such as cyclosporine, leflunomide, and tretinoin may serve as potential therapeutic agents for TIMM8A inhibition [29]. A study by Xiaoyu Zhu et al. also showed that TIMM8A is significantly associated with poor prognosis in both breast cancer and uterine corpus endometrial carcinoma (UCEC), which may mediate tumor immune suppression by regulating Th2 CD4+ T cell infiltration, thereby affecting the efficacy of immunotherapy [18].

In our study, we found that TIMM8A was highly expressed in breast cancer tissues and correlated with poor prognosis, which was consistent with previous ones. Sh-TIMM8A inhibited the proliferation and migration of breast cancer cells and promoted cell apoptosis. These in vitro results further confirmed that TIMM8A functions as an oncogene in breast cancer, and silencing its expression effectively suppresses the malignant phenotypes of breast cancer cells. However, the specific molecular mechanism by which TIMM8A regulates breast cancer progression remains largely unclear and requires further investigation. To investigate potential protein–protein interactors (PPIs) of TIMM8A, we used the STRING database to screen for candidate proteins and found that HSPA9 may be the possible one, which is also upregulated in breast cancer. Furthermore, co-immunoprecipitation (Co-IP) assays verified an endogenous interaction between TIMM8A and HSPA9 in stable cells. Functional experiments showed that TIMM8A knockdown markedly declined HSPA9 protein expression but had no significant effect on its mRNA level, suggesting that TIMM8A may regulate HSPA9 protein abundance at the post-translational modification level.

Ubiquitination is one of the most important post-translational modifications (PTMs). The ubiquitin–proteasome system (UPS) controls a wide range of cellular processes by mediating protein degradation and plays a critical role in breast cancer tumorigenesis and progression. Based on the above observations, we hypothesized that HSPA9 protein levels might be modulated by ubiquitination. Using the Degpred database, we identified RNF4 as a potential E3 ubiquitin ligase for HSPA9, and Co-IP assays indeed further validated the interaction between HSPA9 and RNF4. We propose the following mechanism: TIMM8A and RNF4 competitively bind to HSPA9. In breast cancer, loss of TIMM8A increases the binding of RNF4 to HSPA9, thereby promoting the ubiquitination and degradation of HSPA9, weakening HSPA9 protein stability, and ultimately suppressing the malignant progression of breast cancer. In BT-549 and MCF-7 cells, Co-IP assays further confirmed the interaction between HSPA9 and RNF4. Cycloheximide (CHX) experiments and treatment with the proteasome inhibitor MG132 revealed that TIMM8A knockdown accelerates HSPA9 protein degradation, and this process is associated with increased ubiquitination levels. TIMM8A knockdown leads to reduced HSPA9 stability, which subsequently downregulates the phosphorylation levels of the PI3K/AKT pathway. Notably, overexpression of HSPA9 in TIMM8A knockdown cells significantly restores PI3K/AKT pathway activity and partially reverses the inhibitory effects on cell proliferation and migration. In vivo tumor xenograft experiments further demonstrated that TIMM8A knockdown significantly suppresses tumor growth and reduces p-PI3K and p-AKT levels. And we also found that expression of TIMM8A, HSPA9, and Ki-67 was reduced in the sh-TIMM8A group by immunohistochemical analysis. In summary, this study extends previous reports by further elucidating the pro-tumorigenic mechanism of TIMM8A in breast cancer, revealing a novel molecular regulatory network centered on the TIMM8A/HSPA9/PI3K-AKT axis, and providing new insights into the mechanisms underlying breast cancer progression.

Nevertheless, this study has certain limitations. First, the clinical breast cancer sample size included in this study is relatively limited, and we were unable to perform detailed stratified analyses based on clinical molecular subtypes. Therefore, the prognostic value and functional differences of TIMM8A across distinct breast cancer subtypes remain unclear. Second, previous studies suggest that TIMM8A, as a mitochondria-associated protein, may participate in the regulation of the tumor immune microenvironment and may have a potential role in predicting immunotherapy efficacy. However, this study did not explore or validate this direction in depth. Based on these limitations, future studies should expand the sample size and conduct molecular subtype-stratified analyses. Third, in mechanism, while our Co-IP data suggest that TIMM8A and RNF4 may share overlapping or allosterically coupled binding sites on HSPA9, direct evidence from in vitro competition assays using recombinant proteins is required to definitively establish a competitive binding model. Future studies employing purified proteins will be necessary to resolve this mechanism. Additionally, the role and mechanism of TIMM8A in breast cancer immunotherapy response and chemotherapy resistance warrant further investigation, which may provide a more substantial experimental basis for its clinical translation.

## 4. Methods

### 4.1. Bioinformatics Analysis

In this study, the Xiantao (https://www.xiantaozi.com/ (accessed on 1 July 2026), version: 1.0) academic platform, based on the TCGA database and presenting the results in a visualized manner, was conducted for bioinformatics analysis. This platform performs a wide range of analyses, including pan-cancer analysis, differential expression assessment, clinical correlation evaluation, functional clustering, immune function prediction, and related statistical analyses. Transcriptomic data are log2(TPM + 1) transformed before analysis. Samples with normal tissue or incomplete clinical annotations were excluded, and paired samples were used for comparison. In addition, Kaplan–Meier analysis was also performed for overall survival. A *p* value < 0.05 was considered statistically significant. The hazard ratio (HR) and its 95% confidence interval (CI) were calculated based on RNA sequencing data to evaluate the impact of TIMM8A expression on cancer prognosis. The STRING database (https://string-db.org (accessed on 1 July 2026), version: 12.0) was used to construct a PPI network at a medium confidence threshold of 0.4, involving 20 proteins. The Degpred database (http://degron.phasep.pro/ (accessed on 1 July 2026), 2022-05-11version) was employed to find E3 ligases for HSPA9 ubiquitination.

### 4.2. Tissue Microarray (TMA) and Immunohistochemistry (IHC)

The expression of TIMM8A in breast cancer was evaluated using a tissue microarray (TMA) containing pairs of tumors and matched paracancerous tissues of 52 patients’ obtained from our center, and tissue microarrays were constructed by the Yibeirui company. This study was conducted in accordance with the Declaration of Helsinki. This study was approved by the Institutional Ethics Committee of Shanghai Cancer Center (project identification code: 050432-4-1911D) on 4 November 2019.

Briefly, the TMA slide was deparaffinized, rehydrated, and subjected to heat-induced antigen retrieval. Following endogenous peroxidase quenching and serum blocking, the sections were incubated with a primary anti-TIMM8A antibody (1:200; Boster, Cat# A07659-1, Wuhan, China) at 4 °C overnight, visualized with an HRP-conjugated secondary antibody and DAB, and counterstained with hematoxylin. Immunoreactivity was quantified by two independent pathologists using a semi-quantitative scoring system (0–12) based on background-subtracted digital images. The final score was calculated by multiplying the staining intensity score (0, negative; 1, light yellow; 2, brownish yellow; 3, dark brown) by the positive cell percentage score (0, 0%; 1, 1–25%; 2, 26–50%; 3, 51–75%; 4, ≥75%). Based on the total scores, TIMM8A expression was categorized as negative (0), weak (1–4), moderate (5–8), or strong (9–12).

### 4.3. Cell Culture

HBL-100, MDA-MB-231, MDA-MB-468, SUM149PT, MDA-MB-453, BT549, MCF-7 and HEK293T cell lines were obtained from the American Type Culture Collection (ATCC) or China Cell Bank. The cells were cultured in corresponding DMEM, L15 or RPMI-1640 medium (Gibco, Grand Island, NE, USA) containing 10% fetal bovine serum (Gibco, Grand Island, NE, USA) and 1% (*v*/*v*) penicillin/streptomycin (Gibco, Waltham, NY, USA). The BT549 cell line was grown in DMEM medium above with 1.1 μg/mL insulin (Beyotime). All cells were incubated at 37 °C in a humidified 5% CO2 environment.

### 4.4. Cell Transfection and Lentiviral Transduction

The short hairpin RNA sequences targeting TIMM8A (sh-#1: 5′-TTTCATCGAGGTAGAGACTCA-3′; sh-#2: 5′-GTGCACCAGATGACTGAACTT-3′; sh-#3: 5′-GTGCGGAGTTCGTCTCTGCAA-3′) were individually inserted into the BR-V108 vector. These recombinant plasmids were mixed with the packaging vectors pMD2G and psPAX2 at appropriate ratios and transfected into HEK293T cells at 80–90% confluence using polyethylenimine (PEI, FUSHENBIO, FSF0002, Shanghai, China). A total of 8 μg of plasmids were co-transfected into HEK293T cells in a 60 mm culture dish. The supernatant was collected 48 h post-transfection and filtered through a 0.45 μm pore-size filter. The virus-containing supernatant was mixed with fresh culture medium at a 1:1 ratio and then added to infect BT549 and MCF-7 cells. Polybrene (Sigma-Aldrich, H9268, St. Louis, MO, USA) was added at a concentration of 8 μg/mL to enhance infection efficiency. Fresh medium was replaced 12 h after infection. After culturing for an additional 3 days, the infection efficiency was assessed by Western blot and real-time PCR.

### 4.5. RNA Preparation, RT-PCR, and Quantitative PCR (qPCR) Analysis

Total RNA was extracted from cells using TRIzol reagent (Sigma, T9424-100mL, St. Louis, MO, USA) and reverse transcribed into complementary DNA (cDNA) using HiScript QRT Supermix for qPCR (Vazyme, R123-01, Nanjing, China). qRT-PCR was performed according to the manufacturer’s instructions for the AceQ qPCR SYBR Green Master Mix (Vazyme, Q111-02, Nanjing, China) to evaluate mRNA expression levels. The primer sequences used in this experiment were as follows: GAPDH (forward: TGACTTCAACAGCGACACCCA, reverse: CACCCTGTTGCTGTAGCCAAA); TIMM8A (forward: GCAGTTGCAGCATTTCATCG, reverse: TCCATGCACTTCTCCCAACAA); HSPA9 (forward: CTATCGCTCCATGCCAAAA, reverse: ATGCCACCCACAAGAATCAC). Relative quantification analysis was performed using the formula F = 2 − ΔΔCt, where ΔCt = Ct(target gene) − Ct(reference gene). −ΔΔCt = mean ΔCt of the control group − ΔCt of each sample, and 2 − ΔΔCt represents the relative expression level of the target gene in each sample compared with that in the control group.

### 4.6. Cell Lysis Western Blot (WB)

BT549, MCF-7 and HBL-100 cells were washed twice with iced PBS and lysed, and then total protein was extracted. Protein concentration was determined using a BCA Protein Assay Kit (HyClone-Pierce, 23225, Waltham, MA, USA). SDS sample loading buffer (5×) was added, and the samples were boiled for 5 min to denature the proteins. Then, 20 μg of protein was subjected to SDS-PAGE. The proteins were subsequently transferred onto a PVDF membrane, which was then blocked with 1× TBST containing 5% skim milk at room temperature for 1 h. The PVDF membrane was incubated with primary and secondary antibodies at room temperature for 1 h, followed by three washes with 1× TBST for 10 min each. Protein bands were visualized using an Immobilon Western Chemiluminescent HRP Substrate Kit (Millipore, WBKLS0100, Billerica, MA, USA), and chemiluminescence was detected with a chemiluminescence imager (GE) to capture the protein bands.

### 4.7. Antibodies

Antibodies were obtained from the following sources: Goat Anti-Rabbit (A0208, 1:1000, Beyotime, Shanghai, China) and Goat Anti-Mouse (A0216, 1:1000) were purchased from Beyotime; Rabbit-mAb TIMM8A (A07659-1, 1:1000), rabbit-mAb HSPA9 (14887-1-AP, 1:10,000) mouse-mAb GAPDH (60004-1-lg, 1:100,000), rabbit-mAb RNF4 (17810-1-AP, 1:1000), mouse-mAb β-actin (66009-1-1g, 1:20,000), rabbit-mAb AKT (10176-2-AP, 1:2000), and mouse-mAb p-AKT (66444-1-1g, 1:4000) were purchased from Proteintech (Wuhan, China); Rabbit-mAb PI3K (4257S, 1:1000) was from CST (Beverly, CA, USA); Rabbit-mAb p- PI3K (bs-3332R, 1:1000, Wuhan, Shanghai) was bought from Biose; and Rabbit-mAb KI-67 (ab16667, IHC 1:200) was from Abcam (Cambridge, UK).

### 4.8. Cell Proliferation Assay

Cells were seeded into white 96-well plates (Corning, 3596) at a density of 1500 cells per well, with three replicates per group. Detection began on the second day after seeding, when 10 μL of CCK-8 reagent was added to each well and incubated for 2 h. The plate was then placed on a microplate reader (Tecan Infinite M200pro, Männedorf, Switzerland) and shaken for 2 min. The optical density (OD) was measured at 450 nm, with cell-free wells used as blank controls. Cell proliferation was quantified based on the absorbance values.

### 4.9. Apoptosis Measurements by Flow Cytometry

Apoptosis was analyzed using the Annexin V-FITC Apoptosis Detection Kit (eBioscience, 88-8007-74, San Diego, CA, USA) according to the manufacturer’s instructions. Briefly, cells were collected, washed twice with pre-chilled PBS, and resuspended in 1× binding buffer at a density of 1 × 10^6^ cells/mL. A 200 μL aliquot of the cell suspension was transferred to a 15 mL culture tube. Then, 5 μL of Annexin V and 5 μL of PI staining solution were added, gently mixed, and incubated for 15 min at room temperature in the dark. Subsequently, 300 μL of 1× binding buffer was added to each tube, and the samples were immediately analyzed by flow cytometry (Millipore, Billerica, MA, USA, Guavaeasy Cyte HT). All experiments were performed in triplicate.

### 4.10. Colony Formation Assay

Cells were digested with trypsin, counted, and seeded into six-well plates at a density of 500 cells per well. Three replicate wells were set for each experimental group. The cells were cultured in an incubator at 37 °C with 5% CO_2_. After 14 days of culture, the medium was discarded, and the cells were washed with PBS. Subsequently, the cells were fixed with 4% paraformaldehyde (Sinopharm Group, Shanghai, China) at room temperature for 30 min and then stained with Giemsa stain (Shanghai Dingguo Biotechnology Co., Ltd., Shanghai, China, AR-0752) for 30 min. After staining, the staining solution was discarded, and the cells were washed twice with ddH_2_O. The plates were air-dried and photographed, and the number of colonies was counted.

### 4.11. Wound Healing Assay

Cells in the logarithmic growth phase were trypsinized, resuspended in complete medium, and seeded into 24-well plates. After overnight culture, cells were allowed to grow to 95–100% confluence. A linear wound was created across the cell monolayer using a yellow pipette tip, and the cells were washed twice with PBS (ServiceBio, G0002, Wuhan, China). Wound healing was monitored at 0 h and 24 h or 48 h using a Cellomics ArrayScan VT1 cell imaging multi-mode microplate detection system (Thermo Fisher Scientific, Pittsburgh, PA, USA).

### 4.12. Cell Migration Assay

Transwell chambers with 8.0 μm pore size polycarbonate membranes (Corning, Corning, NY, USA) were placed in 24-well plates. A total of 5 × 10^4^ cells suspended in 200 μL of serum-free medium were added to the upper chamber, while 600 μL of medium containing 20% fetal bovine serum (FBS) was added to the lower chamber as a chemoattractant to induce cell migration. After incubation at 37 °C with 5% CO_2_ for 12 h, the cells on the membrane were fixed with 4% paraformaldehyde for 15 min, then stained with 0.25% crystal violet for 20 min. Non-migratory cells on the upper side of the membrane were gently removed with a cotton swab. Migrated cells on the lower side of the membrane were counted under an optical microscope at ×200 magnification, and five random fields of view were selected for counting per sample.

### 4.13. Co-Immunoprecipitation (Co-IP)

MCF-7 and BT549 cells were washed twice with pre-chilled PBS, then lysed on ice for 20 min using pre-chilled IP lysis buffer. The cells were transferred into EP tubes, centrifuged, and the supernatants were collected. Protein concentrations were determined using the BCA method. A total of 1.0 mg of protein was taken, and the corresponding antibody was added, followed by overnight incubation at 4 °C with rotation. Meanwhile, 20 µL of beads were washed with PBS and centrifuged, and the supernatant was discarded. The overnight-incubated protein–antibody mixture was then added to the beads and incubated for 1 h at 4 °C. After incubation, the mixture was centrifuged, the supernatant was discarded, and the protein–antibody–bead complexes were washed twice with IP lysis buffer. An appropriate volume of IP lysis buffer and 5× loading buffer were added to the complexes, followed by heating at 100 °C for 5 min. Subsequently, SDS-PAGE was performed, and the subsequent steps were carried out as in a standard Western blot. The antibody information is detailed in the antibody record.

### 4.14. Assessment of Protein Stability

To evaluate the effect of stable knockdown or overexpression of protein on HSPA9 stability, stable, transfected cells were treated with the protein synthesis inhibitor cycloheximide (CHX). Cell proteins were harvested at 0, 2, 4, and 6 h after CHX treatment, and then Western blot analysis was performed to detect changes in HSPA9 protein levels. The information on the primary and secondary antibodies used in this experiment is provided in the antibody record.

### 4.15. Ubiquitination Assay

To assess the ubiquitination level of the target protein, stable transfected cells were treated with the proteasome inhibitor MG132 for 24 h, and then the cells were harvested. Cells were lysed with pre-chilled IP lysis buffer on ice, and protein concentrations were determined by BCA assay. A total of 20 μg of protein was taken for detection of HSPA9, and 1.0 mg of total protein was incubated with HSPA antibody overnight at 4 °C with rotation. The next day, 20 μL of pre-washed beads were added and incubated for 2 h at 4 °C. The bead–antibody–protein complexes were washed three times with pre-chilled IP lysis buffer. An appropriate volume of IP lysis buffer and 5× loading buffer were then added, and the samples were incubated at 100 °C for 5 min. Subsequently, SDS-PAGE was performed, and after transfer, Western blot analysis was carried out using ubiquitin primary and secondary antibodies. Antibody information is detailed in the antibody record.

### 4.16. Tumor Xenograft Model

Female BALB/c nude mice aged approximately 6 weeks were purchased from GemPharmatech Co., Ltd. (Nanjing, China). All animal experiments were approved by the Institutional Animal Care and Use Committee of Fudan University Shanghai Cancer Center (approval No. FUSCC-IACUC-S2026-0212). BT549 ShCtrl and ShTIMM8A cells were resuspended in PBS at a concentration of 1 × 10^7^ cells/200 μL and injected into the mammary fat pads of the mice. Approximately 4 weeks after cell inoculation, tumor length and width were measured every 4 days, and tumor volume was calculated using the formula V = (L × W^2^)/2. On day 42 post-inoculation, the mice were euthanized, and the tumors were excised and weighed. Subsequently, the expression of Ki67 protein in tumor tissues from each group was detected by immunohistochemical staining.

### 4.17. Statistical Analysis

All data were expressed as mean ± standard deviation (SD). Statistical analyses were performed using SPSS 27.0 software, and graphs were generated using GraphPad Prism 10.0 software. Comparisons between two groups were conducted using independent-samples t-test, while comparisons among three or more groups were performed using one-way analysis of variance (ANOVA). Fisher’s exact test was applied for analysis the difference expression of TIMM8A between breast cancer tissues and adjacent tissues, A *p*-value < 0.05 was considered statistically significant.

## 5. Conclusions

In summary, this study demonstrates that TIMM8A is upregulated in breast cancer and promotes cancer cell proliferation, migration, and tumor growth. Mechanistically, the downregulation of TIMM8A leads to increased competitive binding between RNF4 and HSPA9, thereby reducing the stability of HSPA9. The subsequent decrease in HSPA9 levels results in reduced phosphorylation of the PI3K/AKT axis, which in turn inhibits the malignant progression of breast cancer. These findings suggest that TIMM8A holds promise as a novel potential therapeutic target for breast cancer, providing a theoretical basis and experimental foundation for the development of new targeted therapy strategies for breast cancer.

## Figures and Tables

**Table 1:** Expression patterns in breast cancer tissues and para-carcinoma tissues revealed in immunohistochemistry analysis.

TIMM8A Expression	Tumor Tissue		Para-Carcinoma Tissue		*p* Value
	Cases	Percentage	Cases	Percentage	
Low	15	48.4%	51	100%	*p* < 0.001
High	16	51.6%	0	0%	

*p* < 0.05 was considered statistically significant.

## Data Availability

The data used and analyzed during the current study are available from the corresponding author on reasonable request.
